# Spider venom peptides as potential drug candidates due to their anticancer and antinociceptive activities

**DOI:** 10.1590/1678-9199-JVATITD-14-63-18

**Published:** 2019-06-03

**Authors:** Ting Wu, Meng Wang, Wenfang Wu, Qianxuan Luo, Liping Jiang, Huai Tao, Meichun Deng

**Affiliations:** 1 Department of Biochemistry and Molecular Biology, School of Life Sciences, Central South University, Changsha, Hunan 410013, China.; 2 Xiangya School of Medicine, Central South University, Changsha, Hunan 410013, China.; 3 Department of Parasitology, Xiangya School of Medicine, Central South University, Changsha, Hunan 410013, China.; 4 Department of Biochemistry and Molecular Biology, Hunan University of Chinese Medicine, Changsha, Hunan 410208, China.

**Keywords:** spider venom peptides, antitumor, pain, drug candidates

## Abstract

Spider venoms are known to contain proteins and polypeptides that perform various functions including antimicrobial, neurotoxic, analgesic, cytotoxic, necrotic, and hemagglutinic activities. Currently, several classes of natural molecules from spider venoms are potential sources of chemotherapeutics against tumor cells. Some of the spider peptide toxins produce lethal effects on tumor cells by regulating the cell cycle, activating caspase pathway or inactivating mitochondria. Some of them also target the various types of ion channels (including voltage-gated calcium channels, voltage-gated sodium channels, and acid-sensing ion channels) among other pain-related targets. Herein we review the structure and pharmacology of spider-venom peptides that are being used as leads for the development of therapeutics against the pathophysiological conditions including cancer and pain.

## Background

Spiders are air-breathing arthropods belonging to the subphylum Chelicerata and have been on Earth for at least 300 million years [1]. Spiders are one of the most successful venomous animals and, with the exception of predatory beetles, they are the most abundant terrestrial predators that can cause detrimental effects on humans from their venomous bite [2]. Some spider species possess chelicerae that are strong enough to penetrate the human skin during a bite, followed by injection of venom into the body [3]. Spider envenomation can become a public health hazard by causing pain, swelling, diaphoresis, hypertension, patchy paralysis around the site of bitten area and other systematic symptoms [4]. Although a spider bite rarely causes death, extreme discomfort is a common result.

On the other hand, spiders are also potentially beneficial to humans given that a great number of their peptides present some potential therapeutic applications due to their anticancer and analgesic activities. Compounds identified in recent years from spider venoms include polypeptide toxins that contain a specific structural motif known as an inhibitor cystine knot (ICK). Pharmacologically, these toxins have been reported to exert a variety of neuroprotective, antimicrobial, anticancer and antinocieptive effects [5-9]. Furthermore, antimicrobial peptides, which are short proteins, have also been the focus of research over the past two decades [10-14]. Spider peptide toxins have also been recognized as useful agents for their anticancer, hemolytic, analgesic and antiarrhythmic properties [15-18]. In this review, we focus on the potential therapeutic applications and mechanisms of spider peptides, particularly with respect to their anticancer and antinociceptive activity (Table 1).

**Table 1.** Some examples of spider venom peptides as potential drug candidates with anticancer and antinociceptive properties.

	Peptide name	Spider	Amino acids	cell/target mechanisms	Action mechanism/ types of pain	Ref
Antitumor activity	PcTx-1	*Psalmopoeus cambridgei*	40	malignant glioma cells	elevation of CKI proteins by activating ERK1/2 after inhibiting the ASIC	[29-32]
	Lycosin-I	*Lycosa singoriensis*	24	Hela, A549, H1299, HT1080 HepG2, DU145, HCT116 JB6 epidermal cells	elevation of CKI proteins and activation of mitochondrial apoptosis pathway	[35]
	Latarcin 2a	*Lachesana tarabaevi*	26	K562 cells	inactivation of mitochondria and intracellular high osmotic pressure by entry and internalization of latarcin2a	[38, 39]
Antinocic-eptive activity	HWTX-XVI	*Phoneutria nigriventer*	39	N-type VGCCs	inflammatory, mechanical and thermal pain	[49]
	Tx3-6	*Phoneutria nigriventer*	55	N-type VGCCs TRPA1 channel	inflammatory, neuropathic, cancer-related pain mechanical, cold and spontaneous nociception	[58, 59] [63]
	Tx3-5	*Phoneutria nigriventer*	45	L-type VGCCs	mechanical and cancer-related pain	[57, 64]
	Tx3-3	*Phoneutria nigriventer*	34	P/Q- and R-type VGCCs	neuropathic pain	[70,71]
	HNTX-IV	*Ornithoctonus hainana*	35	Nav1.7 channel	inflammatory and neuropathic pain	[79]
	HWTX-IV	*Ornithoctonus huwena*	35	Nav1.7 channel	inflammatory and neuropathic pain	[84]
	ProTX-II	*Thrixopelma pruriens*	30	Nav1.7 channel	inflammatory and neuropathic pain	[7, 48, 89]
	PcTx1	*Psalmopoeus cambridgei*	40	ASIC1a channels	thermal, mechanical, chemical, inflammatory and neuropathic pain	[103, 104]
	PnPP-19	*Phoneutria nigriventer*	19	opioid, cannabinoid receptors NO/cGMP/K_ATP_	inflammatory pain	[118, 119] [120]
	δ-CNTX-Pn1a	*Phoneutria nigriventer*	48	opioid, cannabinoid receptors	inflammatory and neuropathic pain	[122]
	Tx3-1	*Phoneutria nigriventer*	41	cholinergic system	neuropathic pain	[123]
	PT1	*Geolycosa sp*	35	P2X3 receptor	inflammatory pain	[124]

Abbreviations: ASIC: Acid-sensitive ion channel; CKI: Cyclin-dependent kinase inhibitor; HWTX-XVI: Huwentoxin-XVI; HNTX-IV: Hainantoxin-IV; HWTX-IV: Huwentoxin-IV; NO/cGMP/KATP: Nitric oxide/cyclic guanosine monophosphate/ATP-sensitive potassium channel; PcTx1: Psalmotoxin1; PT1: Purotoxin-1; TRPA1 channel: Transient receptor potential cation channel subfamily A member 1; Tx: Toxin; VGCCs: Voltage-gated calcium channels.

### Antitumor peptides from spider venom

Cancers have become the leading cause of death around the world and have a significant impact on public health [19]. In 2012, an estimated 14.1 million new cancer cases were diagnosed while 8.2 million died of the disease worldwide. The National Cancer Institute reported that in 2016, there were about 1.7 million new cancer cases in the United States and 0.6 million cancer deaths [20]. Over the past few decades, significant advancements in the treatment of cancer have included chemotherapy, radiation therapy, immunotherapy and surgery. Nevertheless, major problems of these conventional therapies include a relatively low success rate, serious side effects and drug resistance [21, 22]. Therefore, an unmet need in this field is to develop treatments for cancer patients that are more effective and less toxic. In recent years, a large number of studies have shown that biotoxins - which are produced by snakes, spiders, bees, scorpions, wasps and ants - have anti-tumor potential [23-26]. The peptides isolated from their venoms can impair tumor cell membrane, inhibit cancer cell growth or induce apoptosis [27, 28]. Elucidation of the anti-tumor effect of spider venom is still in its infancy although its pharmacological activity is well documented. Spider venom peptides have been confirmed as killing tumor cells or inhibiting their proliferation. At the same time, many experiments have detected the cytotoxicity of spider peptides to normal cells and the results are encouraging.

**Figure 1.**
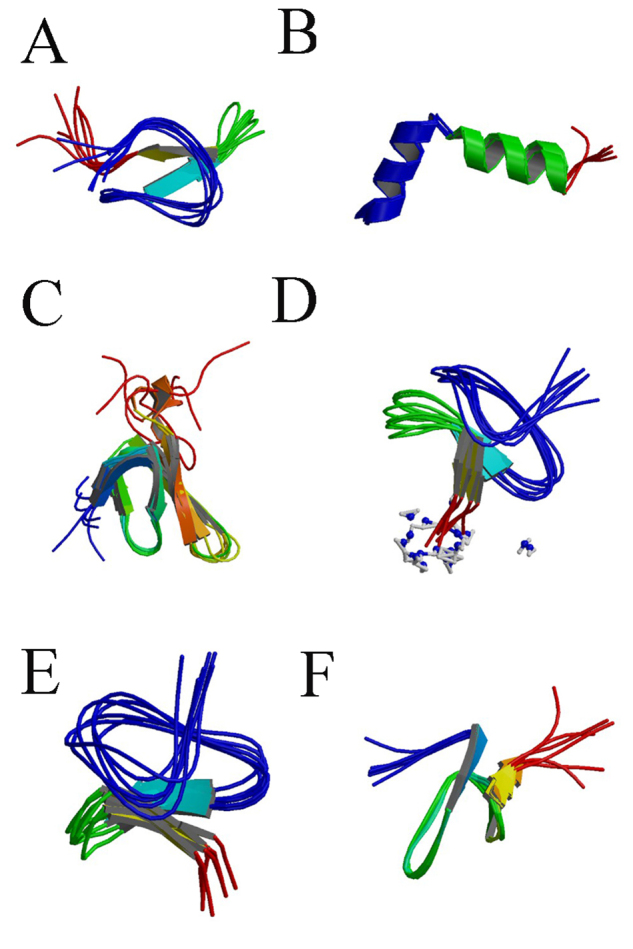
**The 3D structure of spider venom peptides.** (**A**), PcTx1(Psalmotoxin1,PDB ID: 1LMM); (**B**), Latarcin 2a (PDB ID: 2G9P); (**C**), ω-agatoxin-IVA (PDB ID: 1IVA); (**D**), HNTX-IV (Hainantoxin-IV, PDB ID: 1RYG); (**E**), HWTX-IV (Huwentoxin-IV, PDB ID: 1MB6); (**F**), ProTx-II (PDB ID: 2N9T).

**Figure 2.**
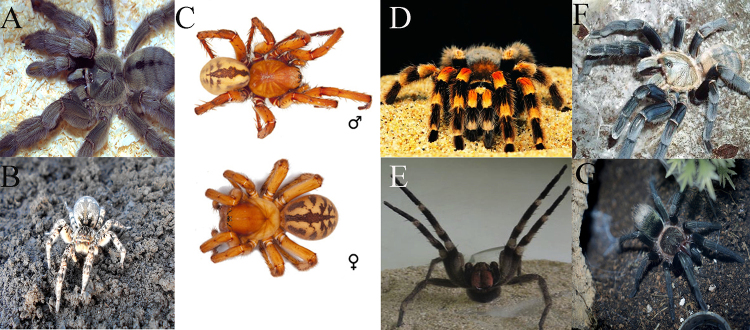
**Pictures of spiders.**(**A**), *Psalmopoeus cambridgei,* the picture was provided by Micha L Rieser; (**B**), *Lycosa singoriensis,* the picture was provided by Avereanu*;* (**C**), *Lachesana tarabaevi,* the picture was provided by Alexey *et al.* [40] ; (**D**), *Ornithoctonus huwena;* (**E**) *Phoneutria nigriventer,* the picture was provided by Peigneur *et al.* [56]; (**F**), *Ornithoctonus hainana;* (**G**), *Thrixopelma pruriens,* the picture was provided by Vanessa S.

**Figure 3.**
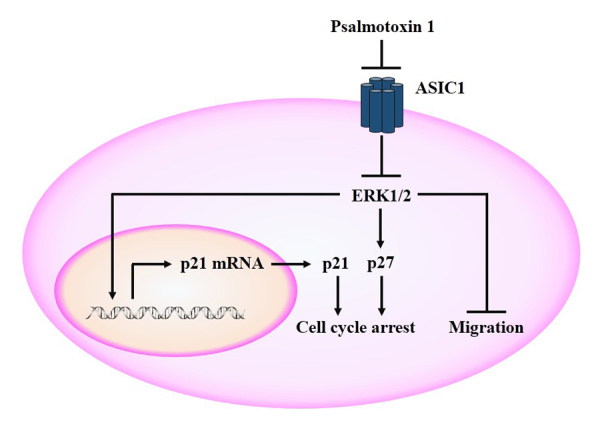
**The anti-tumor mechanism of PcTx1.** PcTx1 can suppress activation of ERK1/2 by inhibiting ASIC1 channels, followed by upregulating p21 and p27 protein expression to arrest the cell cycle. The level of p21 mRNA increases, but that of p27 mRNA does not change. The inhibition of ERK1/2 phosphorylation also restricts migration, but the mechanism remains unclear. ASIC1: Acid-sensitive ion channel 1; PcTx1: Psalmotoxin1.

Psalmotoxin 1(PcTx1) (Figure 1A) is a specific acid-sensitive ion-channel-1 (ASIC1) blocker from the venom of the South American tarantula *Psalmopoeus cambridgei* (Figure 2A). PcTx1 effectively inhibited basally active cation currents in malignant astroglioma cells [29, 30]. Similar results were observed in freshly resected glioblastoma multiforme (GBM) cells but not in normal human astrocytes. These results are encouraging because gliomas are highly invasive while ion transport can play a critical role in both migration and proliferation of glioma cells [31]. PcTx1 may be a good candidate that can improve the currently poor prognosis for the treatment of patients with glioblastoma multiforme. Subsequently, Arun K *et al.* also found that PcTx1 arrests the cell cycle in the G0/G1 phase and upregulates the expression of two proteins, p21 and p27, due to a reduction of the phosphorylation of ERK1/2. Similar results were also observed in other lines of GBM cells (Figure 3) [32]. Interestingly, glioma patients often suffer from epileptic seizures, which are due to neurotransmitter release from the tumor cells [33]. In normal cells, the release of neurotransmitters is controlled by membrane depolarization events. Similarly, electrical bursting activity in brain tumor cells may be also be induced by extracellular pH change that enhances Na^+^ ion flux through the PcTx1-sensitive ASICs [34]. These findings demonstrate that PcTx1 may be useful as a drug candidate to reduce the occurrence rate of epileptic seizures in glioma patients.

**Figure 4.**
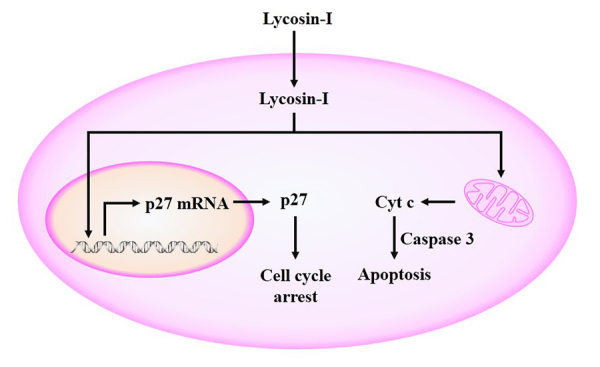
**The anti-tumor mechanism of Lycosin-I.** Lycosin-I penetrates into cytoplasm and upregulates p27 mRNA and protein expression to inhibit the cell cycle. Lycosin-I can bind with mitochondria membrane and promote a mitochondria-mediated death pathway to induce tumor cell apoptosis. Cyt c: cytochrome c.

Lycosin-I is a cationic peptide isolated from *Lycosa singoriensis* (Figure 2B). There is sufficient evidence to prove that lycosin- I can inhibit tumor growth *in vitro* and *in vivo* by activating dual signaling pathways, including inhibition of proliferation and induction of apoptosis. Treatment with lycosin- I (40 μM) resulted in more than 90% cell death in the following human tumor cell lines: colon adenocarcinoma (HCT-116), cervix carcinoma (HeLa), fibrosarcoma (H1080), hepatocellular carcinoma (HepG2), lung adenocarcinoma (H1299, A549), and prostate carcinoma (DU145). In contrast, the same dose of lycosin-I was less toxic to non-tumor cells. Lycosin- I activated the mitochondria-mediated death pathway to sensitize cancer cells for apoptosis, and upregulated p27 to inhibit cell proliferation (Figure 4) [35]. In order to produce effects, it is necessary for lycosin-I to bind and penetrate the cell membrane. Besides the electrostatic attraction between tumor cell membranes and lycosin-I, Tan *et al.* found that lycosin-I gradually aggregated upon contacting the lipid membrane, followed by its absorption and structural change, which reduced its diffusion dynamics. This new insight on lycosin-I may help us understand how lycosin-I interacts with the tumor cell membrane [36]. To enhance the cellular entry and efficacy in solid tumor, the method of substituting one amino acid (from Lys to Arg) was utilized to design a synthetic cationic peptide (Lycosin- I to R- Lycosin-I). Compared with lycosin-I, R-lycosin-I demonstrated higher inhibitory activity and selectivity toward cancer cells [37]. Based on its characteristics mentioned above, lycosin- I is recognized as a cell-penetrating peptide that can be used for the intracellular delivery of functional materials to circumvent the biomembrane barrier by conjugating with spherical gold nanoparticles. This conjunction will provide a stable, efficient and higher selective platform to diagnose and treat cancers correctly in the future [6, 37].

**Figure 5.**
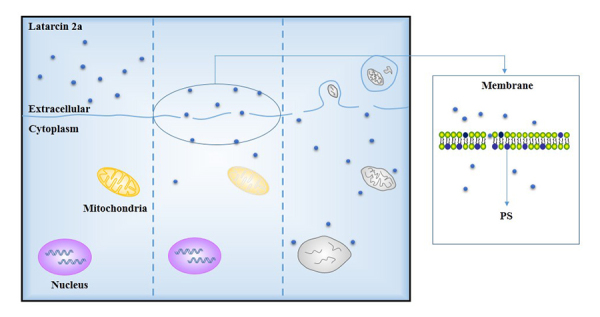
**The mechanism of Latarcin 2a interaction with K562 cells.**Latarcin 2a bound to membrane and small pores reform in cells membrane. The latarcin 2a penetrated into cytoplasm, which induced inactivation of mitochondria and externalization of PS. There was a positive feedback between externalization of PS and internalization of latarcin 2a. At last, the cell disintegrated due to the high intracellular osmotic pressure caused by accumulation of the peptide. PS: Phosphatidyl serine.

Latarcin 2a (Figure 1B), a short linear antimicrobial peptide, is purified from the Central Asian spider *Lachesana tarabaevi* (Figure 2C) and displays *in vitro* cytotoxicity against human erythroleukemia (K562) cells. Latarcin 2a induced changes in cell membrane, to which it was closely bound, then rapidly penetrated into the cytoplasm and accumulated in perinuclear structures. Latarcin 2a induced membrane blebbing and swelling of K562 cells followed by cell death. The formation of small pores on the cell membrane during the development of blebs plays an important role in the peptide’s induction of cytotoxicity in K562 cells. Formation of small membrane pores leads to internalization of latarcin 2a, which induces inactivation of mitochondria and externalization of phosphatidylserine (PS). The process was a positive feedback with latarcin 2a internalization and PS externalization further promoting the accumulation of latarcin 2a. When the growth rate of cell and cell membrane through development of blebs did not match the intracellular high osmotic pressure, K562 cells membrane totally disintegrated (Figure 5). Surprisingly, externalization of PS did not induce activation of the apoptosis pathway [38-40].

The venom of the spider *Macrothele raven* has an effect on proliferation and apoptosis of different types of tumor cells. The venom promotes HeLa cell apoptosis and necrosis in a time- and dose-dependent manner. Elevation of caspase-3 activity plays a critical role in apoptosis with complete or partial suppression of tumor size observed *in vivo* in nude mice. Further examination showed that G0/G1 cell cycle arrest may contribute to the inhibitory effect of venom on the growth of HeLa cells [41]. The venom of *M. raveni* also exhibited dose-dependent antitumor activity in human breast carcinoma (MCF-7) cells, inhibiting DNA synthesis, affecting cell viability, and inducing apoptosis and necrosis. The venom arrested the cell cycle in the G2/M and G0/G1 phases and upregulated p21 in the human breast carcinoma cell line MCF-7 [42]. The venom also inhibited the proliferation of myelogenous leukemia cell line K562 and activated caspase 3 and caspase 8 pathway to induce K562 cell apoptosis [43]. In an *in vitro* and *in vivo* experiment, the venom upregulated the expression of PTEN and BAX to inhibit the growth of subcutaneous H_22_ tumors in mice and downregulated PI3K, AKT and mTOR to inhibit tumor cell proliferation [44]. A similar pathway was confirmed in extrahepatic metastatic hepatocellular carcinoma whereas the Bad protein level was also upregulated to antagonize the anti-apoptotic effects of Bcl-2 and induce tumor cell apoptosis [45]. However, it is worth noting that whether the effects of this venom are due to peptides remains unknown and requires further research.

There are different mechanisms involved in anticancer activity of spider peptides. On the one hand, the peptide produces effects by itself through directly increasing intracellular osmotic pressure, as a result of its internalization [38]. On the other hand, the peptides play an important role in inhibiting the growth of tumor cells by regulating downstream signal molecules, especially proliferative and apoptotic pathways. These results suggest that spider peptides may serve as anticancer drug candidates. However, in our point of view, because of its penetrating ability, accumulation near nuclear membrane and effects on transcriptional level, we can hypothesize that some peptides may enter the nucleus and bind with specific DNA sites to regulate transcription. The mechanism should be the focus of future research.

### Antinociceptive peptides from spider venom

Pain is the main reason that persons seek medical care and a major problem that continues to challenge the medical profession. There are several types of pain that seriously affect patients’ quality of life and cause economic burden. It is estimated that chronic pain affects ~15% of the adult population, while the annual economic burden caused by chronic pain is approximately $600 billion in the United States, which exceeds the sum of economic costs caused by cancer, diabetes and stroke [46]. While a variety of drugs, such as non-steroidal anti-inflammatory drugs and opioids, are utilized to prevent and treat different types of pain, they may cause side effects. Moreover, it is reported that chronic opioid treatment leads to development of drug resistance [47, 48]. Therefore, it is an urgent need to seek new antinociceptive drugs to increase efficacy and reduce side effects. Recent studies have indicated that peptides isolated from spiders can function as antinociceptive drugs by acting on ion channels [7, 17, 49].

### Spider venom peptides acting on ion channels as antinociceptive drugs

### Calcium channel inhibitors and analgesia

Pain information processing starts from the activation of peripheral nociceptors, causing action potentials to propagate along the primary afferent nerve fibers into sensory neurons in dorsal root ganglia (DRG). They are further relayed to the spinal dorsal horn through the central axons of sensory neurons. The action potentials reaching the central terminals of sensory afferents cause membrane depolarization, activation of voltage-gated calcium channels (VGCCs), and calcium influx, which triggers synaptic vesicle exocytosis. This then leads to the release of excitatory neurotransmitters including glutamate, pain-inducing peptides such as substance P and calcitonin gene-related peptide (CGRP) into the synaptic cleft. These neurotransmitters then cause activation of post-synaptic dorsal horn projection neurons and interneurons, leading to spinal modulation of sensory signals [50]. Thus, changes in the expression and functions of VGCCs in pain-inducing conditions can be potential targets for pain management. VGCCs comprise a family of ion channels divided into low-threshold (T-type) and high-threshold (L-, N-, P/Q-, and R-type) voltage-gated channels. Based on the sequence homology of the main pore-formed α1 subunit, VGCCs are divided into 3 families: Cav1, Cav2, and Cav3 [51]. VGCCs emerged as potential targets to treat severe pain conditions, as for example by the extraction of a peptide toxin from the venom of the cone snail *Conus magus*, the ω-conotoxin MVIIA, as a treatment for severe pain refractory to other treatments. The ω-conotoxin MVIIA (synthetic form known as ziconotide, Prialt) produces pain relief by blocking N-type VGCC [52-54].

Huwentoxin-XVI (HWTX-XVI), which is purified from the venom of the spider *Ornithoctonus huwena* (Figure 2D), is a specific blocker of N-type VGCCs in rat DRG neurons. HWTX-XVI contains 39 amino-acid residues including six cysteines that form three disulfide bridges. In formalin tests with rats, intraperitoneal injection of HWTX-XVI produced potent dose-dependent antinociceptive effect in phase II but not in phase I. The post-incision pain reduction by intramuscular injection of HWTX-XVI occurred immediately and lasted longer, while morphine-induced pain reduction was also immediate but lasted for a shorter time. Moreover, HWTX-XVI also induced a slight but significant antinociceptive effect on thermal pain but had no effect on the motor coordination of animals without inducing any obvious side effects, such as serpentine tail movements or whole body shaking caused by ziconotide, even at the highest dose tested [49]. The antinociceptive effect and minimal side effects of the toxin demonstrate that HWTX-XVI can be a potent candidate for the control of pain.

The venom from the Brazilian armed spider, *Phoneutria nigriventer* (Figure 2E), includes many peptides that have different functions [55, 56]. *Phoneutria nigriventer* toxin3-6 (Tx3-6), one of the six peptide isoforms of fraction PhTx3 in the venom, preferentially blocks N-type VGCCs [57]. Tx3-6 exhibits an antinociceptive effect on inflammatory and neuropathic pain and has a wide therapeutic window [58]. Tx3-6 also shows a strong and dose-dependent antinociceptive effect on cancer-related pain and causes minimal side at high doses. Besides minimal side effects, Tx3-6 indicated a good tolerability and a higher therapeutic index [59]. These results suggest that additional mechanisms besides N-type VGCCs contribute to its antinociceptive effect. The transient receptor potential cation channel subfamily A member 1 (TRPA1) channel is primarily localized to a subpopulation of primary sensory neurons of the trigeminal, vagal, and DRG. It is activated by reactive endogenous and exogenous substances and noxious cold. This subset of nociceptors produces and releases two neuropeptides, substance P and CGRP, which make TRPA1 channel associate with a series of pains [60-62]. Tx3-6 selectively inhibited calcium responses and currents evoked by the TRPA1 agonist, allyl isothiocyanate (AITC). Low doses of Tx3-6 attenuated acute nociception as well as the mechanical and cold hyperalgesia evoked by AITC [63]. It is apparent that Tx3-6 also produced antinociceptive effects by acting on the TRPA1 channel.

*Phoneutria nigriventer* toxin 3-5(Tx3-5) is a blocker of L-type VGCCs and produces an antinociceptive effect in postoperative models about 180 times more potent than that of ziconotide [64]. This effect was reversed by the selective activator of L-type VGCCs Bay-K8644. Moreover, Tx3-5 was effective in reducing cancer-related pain and could be utilized to treat cancer patients who are resistant to morphine. However, the antinociceptive effect caused by Tx3-5 in neuropathic pain models was limited [57]. Several studies have indicated that the role of L-type VGCCs in neuropathic pain is not as important as those of other types of calcium channels, such as P/Q, N or R-type VGCCs [65-67]. The effect induced by Tx3-5 was related to subtype L-type VGCCs, Ca_v_1.2 [68, 69]. Additionally, Tx3-5 did not cause adverse motor effects at efficacious doses [57]. These results imply that L-type VGCCs can be used as a target for pain relief.

*Phoneutria nigriventer* toxin3-3 (Tx3-3) blocks P/Q- and R-type VGCCs. The peptide caused a short-lasting antinociceptive effect in the nociceptive pain test and a long-lasting antinociceptive effect in neuropathic pain rather than inflammatory pain [70]. More importantly, Tx3-3 indicated a better safety profile when compared to the P/Q-blocker ω-conotoxin MVIIC, which elicits serious motor dysfunctions. It is tempting to speculate that additional affinity of Tx3-3 for R-type VGCCs plays a potential role in nociception but not in motor functions [70]. Furthermore, Tx3-3 exhibited greater inhibitory effects on dorsal horn neuronal responses under neuropathic pain condition compared to normal physiological conditions. However, ω-agatoxin-IVA (Figure 1C), a blocker of spinal P/Q-type VGCCs, attenuates neuronal responses in physiological conditions rather than neuropathic conditions [67]. These results suggest that Tx3-3 inhibits neuropathic pain and that R-type VGCCs a important target for neuropathic pain relief [71].

### Sodium channel inhibitors and analgesia

Nociceptors detect noxious conditions to produce the sensation of pain, and this signal is conveyed to the CNS by means of action potentials. In all electrically excitable cells in mammals, the fast upstroke of action potentials is mediated by voltage-gated sodium channels (VGSCs). Therefore, VGSCs inhibitors are capable of blocking the conduction of an electrical signal and producing an antinociceptive effect [72]. At least nine mammalian subtypes (Nav1.1-1.9) have been identified in the nervous system [73]. Among these, the tetrodotoxin-sensitive (TTX-S) sub-types, Nav1.1, Nav1.6, and Nav1.7, and the TTX-resistant (TTX-R) sub-types, Nav1.8 and Nav1.9, are expressed in adult nociceptive DRG neurons. In light of the genetic evidence that specific VGSCs sub-types are related to specific types of pain, it is now evident clear that Nav1.7 is an important player in the generation of nociceptive sensations in humans [48, 74, 75].

Hainantoxin-IV (HNTX-IV) (Figure 1D), a 35-amino acid peptide purified from the venom of Chinese bird spider, *Ornithoctonus hainana* (Figure 2F), suppressed TTX-S sodium channels current [76]. In this peptide, four residues (Lys27, His28, Arg29 and Lys32) are observed forming a positively charged patch on the molecular surface, which is critical for the inhibitory activity of HNTX-IV by interacting directly with the acidic residues in the DII S3-S4 linker of TTX-S sodium channels and stabilizing the DII S4 voltage sensor [77-80]. HNTX-IV efficiently reversed inflammatory and neuropathic pain. The efficiency of HNTX-IV in both models equivalent to that of morphine. In the spinal nerve model, the antinociceptive effect of HNTX-IV on allodynia was longer and more potent than mexiletine. These results demonstrate that HNTV-IV efficiently alleviates acute inflammatory pain and chronic neuropathic pain and provides an attractive template for further clinical antinociceptive drug design [79]. Besides the Nav1.7 channel, HNTX-IV also blocked other subtypes of VGSCs, such as Nav1.6, which was related to the symptoms of oxaliplatin neuropathy [81]. This may partly explain the presence of side effects caused by HNTX-IV.

Huwentoxin-IV (HWTX-IV) (Figure 1E) is a 35-amino-acid voltage-sensor-gating modifier from the venom of the tarantula species *Ornithoctonus huwena*. The peptide has an inhibitory effect on a TTX-S channel in adult rat DRG neurons with high selectivity towards Nav1.7. HWTX-IV docks at neurotoxin receptor site 4, located at the extracellular S3-S4 linker of domain II in Nav1.7, and traps the voltage sensor of domain II in the inward, closed configuration [82, 83]. HWTX-IV possessed dose-dependent and statistically significant antinociceptive effects on acute inflammatory pain and chronic neuropathic pain. However, the effect of morphine on inflammation pain was 2 times more potent than that of HWTX-IV. Compared to mexiletine, the peptide produced a longer and greater antinociceptive effect on allodynia in the SNI model [84]. These results suggest that HWTX-IV is a potential and efficient candidate for further clinical drug development against inflammatory and neuropathic pain.

ProTx-II (Figure 1F), a 30 amino acid cystine knot peptide, was purified from the venom of the green velvet tarantula *Thrixopelma pruriens* (Figure 2G). ProTx-II traps the domain I, II and IV voltage sensor in the resting configuration and is at least 100-fold more selective towards Na_V_1.7 over other sodium channel subtypes [85]. A patch of amino-acid residues (Lys-4, Trp-5, Met-6, and Trp-7) on the surface of ProTx-II has been identified as promoting lipid binding, which is the first step toward exhibiting an inhibitory effect [86]. ProTx-II suppresses VGSCs by decreasing channel conductance and shifting activation to more positive potentials [87]. Na_V_1.7 may regulate firing thresholds by magnifying generator potentials [88]. As a result, shifting activation to more positive potentials reduces neuronal excitability and pain sensitivity but maintained the ability to sense acute painful stimuli. ProTx-II exhibited a dose-dependent and significant antinociceptive effect on neuropathic and inflammatory pain without any severe effect on motor function [7, 48]. Furthermore, ProTx-II decreases spontaneous action potentials in DRGs occurring in rats with chemotherapy-induced peripheral neuropathy (CIPN) and significantly attenuates behavioral signs of CIPN [89]. Moreover, because of the inability of the peptide to cross the blood-nerve barrier, ProTx-II does not inhibit action potential propagation in intact nerve [87]. These findings indicate that ProTx-II is a potential drug for treating different types of neuropathic pain.

### Acid-sensing ion-channel inhibitors and analgesia

Acidosis occurs in different clinical conditions, such as in inflammation, fractures, lesions and postoperative state. After detecting noxious stimuli, the peripheral free terminals of nociceptive neurons can be depolarized by activating ion channels through protons. This is how an acidic pH can produce algogenic effects. These channels, which can be activated by protons, are divided into two families. The first is Transient Receptor Potential channels that play an important role in the Transient Receptor Potential Vanilloid receptor type 1(TRPV1). The second is ASICs, which are widely expressed in the central and peripheral nervous system. At least nine isoforms (1a-b, 2a-b, 3a-c, 4, 5) encoded by five genes have been detected in mammals [90-92]. ASIC1a, ASIC2a and ASIC2b are widely expressed in the central nervous system [93, 94]. Recent studies have linked ASICs to pain and used them as a target for the treatment of pain [95-97]. Some peptides isolated from spider venoms have been confirmed to regulate ASIC1, including PcTx1, Hi1a, Hm3a, Ma1-3 and MitTxα/β [9, 98-101].

As mentioned above, PcTx1 was able to block ASIC1a in both brain neurons and spinal neurons [29, 30, 102]. It was reported that PcTx1 exhibits potent antinociceptive effects on several types of pain models in rodents. In tail immersion and hot plate tests, PcTx1 induced obvious antinociceptive effects similar to morphine. In the formalin test, PcTx1 produced an evident antinociceptive effect on irritant chemical nociception and inflammation. In a persistent pain model caused by chronic constriction injury of the sciatic nerve in rats, PcTx1 abolished both tactile allodynia and thermal hyperalgesia. Similarly, PcTx1 reversed thermal and mechanical hyperalgesia induced by vincristine. Additional research showed that the antinociceptive effects of PcTx1 and morphine were not addictive and that PcTx1 treatment also produces tolerance but no side effects. PcTx1 produced its antinociceptive effect via stimulating the μ- and δ-opioid receptors. Specific molecular mechanisms need to be revealed by further studies. ASIC1a and Met-enkephalin were co-expressed in the dorsal horn neurons whereas the block of ASIC1a leads to the activation of encephalin system. The specific mechanisms need further study, but may involve ASIC1a in spinal inhibitory interneurons [103]. Thus, in the cerebrospinal fluid, PcTx1 increased encephalins (endogenous opioid peptides), which were potent ligands of both μ-and δ- opioid receptors, followed by the activation of μ-and δ- opioid receptors, leading to antinociceptive effects [104]. Interestingly, it was shown that PcTx1 exerts dual actions on ASIC1a/2a by using a whole-cell patch clamp. It produced inhibitory or enhanced effects, determined by the pH. Potentiation was the strongest at moderate pH by increasing the apparent affinity of channel activation for protons [89]. These findings help to elucidate the diverse and complex pharmacology of PcTx1.

It is obvious that ion channels contribute to treating different types of pain. Compared with traditional analgesic drug, spider peptides act immediately and last for a longer time. Furthermore, the peptides recover the tolerance of morphine, which indicates that the peptides-morphine constitute a good combination for future clinical application [49, 57, 79, 84]. Compared with ziconotide, another animal peptide approved by the Food and Drug Administration, spider peptides possess similar efficacy and fewer side effects, which highlights its indication to be applied early in clinic practice [49, 59]. Beyond that, heart failure has become a common condition. Ion channels perform an enormous function on cardiac electrophysiology, which is the basis of a normal heartbeat. It is speculated that spider peptides, such as GsMTx-4 [15] may promote an immense influence on heart disease.

### Other spider peptides showing antinociceptive effects

Besides the spider peptides acting on ionic channels, probably there are other targets involved in antinociceptive activity of spider venom peptides due to the complex neural mechanisms of nociceptive processing. There are other spider toxins, whose mechanisms are not yet demonstrated or do not seem to involve ionic channels, that will be described herein.

Opioid receptors (including three types, μ, δ and κ receptors) and cannabinoid1 (CB1) receptors are expressed in both the central and peripheral nervous systems. Several studies have demonstrated that endogenous opioids and cannabinoids are involved in the nociceptive pathway and antinociceptive action [105-110]. The nitric oxide/cyclic guanosine monophosphate/ATP-sensitive potassium channel (NO/cGMP/K_ATP_) pathway has been described as being associated with antinociception induced by different analgesic drugs [111-113]. Muscarinic and nicotinic acetylcholine (ACh) receptors are emerging as important targets for the development of novel treatments for chronic pain [114, 115]. Some of peptides isolated from the venom of the spider *Phoneutria nigriventer* or their modified peptide can exert antinociceptive effects through the above mentioned pathways.

PnPP-19, a synthetic and nontoxic peptide designed from *Phoneutria nigriventer* toxin2-6 (PnTx2-6), comprises the potential active core (19 amino acid residues) of the PnTx2-6 [116, 117]. Recently, researchers have proven that PnPP-19 exerts profound influence on antinociceptive functions in both the central nervous system and the peripheral nervous system [118, 119]. PnPP-19 selectively exerts direct activation of μ-opioid receptors by inducing indirect inhibition of calcium channels and thereby impairing calcium influx in DRG neurons [117]. Moreover, the PnPP-19-induced activation of opioid receptors, but did not stimulate the recruitment of β-arrestin2. Besides, PnPP-19 was able to indirectly, activate δ opioid receptors, which may occur though an indirect pathway involved in inhibition of neutral endopeptidase (NEP), an enzyme imperative for the cleavage of many endogenous peptides, thus enhancing the endogenous opioid level to strengthen antinociceptive response [118]. These reports confirm the role of PnPP-19 in the opioid system. Another study reinforces the influence of PnPP-19 on antinociceptive responses and highlights its role in endothelial nitric oxide synthase (eNOS) and neuronal nitric oxide synthase isoforms in the pain pathway [120]. PnPP-19 caused an increase of NO levels via activating both neuronal nitric oxide synthase (nNOS) and eNOS, thus triggering NO/cGMP/KATP pathway.

A 48-amino-acid polypeptide from *Phoneutria nigriventer* spider venom [121], known as δ-CNTX-Pn1a (also called PnTx4(6-1)), whose antinociceptive effects were tested in different pain models, is associated with the activation of μ, δ opioid receptors and CB1 receptors [122]. The deeper mechanisms, which may be similar to those of PnPP-19, have not been further clarified. PhKv (also called Tx3-1), a 4584 Da peptide, was also isolated from the venom of the armed spider *Phoneutria nigriventer*. PhKv can inhibit the Ach enzyme, thus inducing the increase of the ACh content at the neuronal synapses and thereby leading to the activation of the cholinergic system and the antinociceptive response [123].

Purotoxin-1 (PT1) is a novel peptide isolated from the venom of the Central Asian spider *Geolycosa sp.*, and is the first natural molecule found to exert powerful and selective inhibitory action on P2X3 receptors. The peptide demonstrated potent antinociceptive properties on inflammatory pain in animal models [124]. Thus, P2X3 receptors also play a critical role in antinociceptive effects of this spider venom peptide.

It is widely accepted that morphine, as the exogenous μ receptor agonist, can produce central and peripheral antinociceptive effects mediated by activation of CB1 receptors [105, 125], which was also presented by PnPP-19. This finding highlights the tolerability of PnPP-19, which should be further explored. In addition, PnPP-19 probably causes less sides effects due to the non-recruitment of β-arrestin2. Beyond that, we should be fully aware of additional unknown possible mechanisms involved in the pain pathway and also attend to their side effects by comparing it with other drugs.

## Conclusion

Spider venom is a complex mixture comprising a large number of biologically active peptides, enzymes and organic and inorganic compounds. Some of the spider venom peptides directly or indirectly participate in regulating growth and death of tumor cells. They also target the various types of ion channels and other pain pathways to play an important role in antinociceptive responses. These effects make spider peptides potential candidates for drug development. Successful examples of snake venom peptides that manage to reach the market improve confidence to convert spider venom peptides to drugs. The development and application of new methodologies, such as high throughput screening, allow us to ascertain more information on the components of animal venom and to verify their function. By examining the genetic similarity and dissimilarity between spiders and snakes, the modification of spider peptides into clinical versions becomes more efficient. Although no spider venom-derived drug has yet been applied, the situation may be changed completely, if at least one of the spider genomes were to be sequenced. Conjugation of the peptides with polymeric materials, such as gold nanoparticles, is essential not only to solve the problem of cytotoxicity of the toxins but also to obtain modification of specific sites. Therefore, we are confident that the development of modern technology and a better understanding of spider toxin peptides will accelerate the transformation of such peptides into pharmacological leads for the development of novel therapeutic agents and strategies against clinical diseases.

### Abbreviations

ASIC1: Acid-sensitive ion channel 1; AITC: Allyl isothiocyanate; CB1: Cannabinoid1; CGRP: Calcitonin gene-related peptide; CKI: Cyclin-dependent kinase inhibitor; Cyt c: cytochrome c; DRG: Dorsal root ganglia; eNOS: Endothelial nitric oxide synthase; GBM: Glioblastoma multiforme; HWTX-XVI: Huwentoxin-XVI; HNTX-IV: Hainantoxin-IV; HWTX-IV: Huwentoxin-IV;ICK: Inhibitor cystine knot; NEP: Neutral endopeptidase; nNOS: neuronal nitric oxide synthase; NO/cGMP/K_ATP_: Nitric oxide/cyclic guanosine monophosphate/ATP-sensitive potassium channel; PcTx1: Psalmotoxin1; PI: Propidium iodide; PS: Phosphatidyl serine; PT1: Purotoxin-1; SNL: Spinal nerve spinal; TTX-R: Tetrodotoxin-resistant; TRPA1 channel: Transient receptor potential cation channel subfamily A member 1; TRPV1: Transient receptor potential vanilloid receptor type1; TTX-S: Tetrodotoxin-sensitive; Tx: *Phoneutria nigriventer* toxin; VGCCs: Voltage-gated calcium channels; VGSCs: Voltage-gated sodium channels.
